# LPA1 receptor involvement in fibromyalgia-like pain induced by intermittent psychological stress, empathy

**DOI:** 10.1016/j.ynpai.2017.04.002

**Published:** 2017-04-17

**Authors:** Hiroshi Ueda, Hiroyuki Neyama

**Affiliations:** Department of Pharmacology and Therapeutic Innovation, Nagasaki University Graduate School of Biomedical Sciences, Nagasaki, Japan

**Keywords:** Fibromyalgia, Empathy, Psychological stress, Mirtazapine, Morphine, Lysophosphatidic acid

## Abstract

Treatment for fibromyalgia is an unmet medical need and its pathogenesis is still poorly understood. The present study demonstrated that intermittent psychological stress (IPS), or empathy causes generalized chronic abnormal pain with female predominance. The persistence of the pain phenotype was dependent on the unpredictability of the stressor. The pain was reversed by pregabalin (PGB), duloxetine (DLX) or mirtazapine (Mir), but not by diclofenac or morphine. Differential administration of these existing medicines revealed that the sites of PGB and Mir actions exist in the brain, but not in the spinal cord, while that of DLX is preferentially in the spinal cord. It is interesting to note that the intracerebroventricular injection of PGB or Mir showed potent analgesia for 24 h or longer, though systemic injection of these medicines shows anti-hyperalgesia just for several hours. These results indicate that initial intense actions in the target brain may prevent the forthcoming development of pain memory. IPS-induced abnormal pain was prevented in mice deficient of lysophosphatidic acid receptor 1 (LPA1) gene, and completely cured by the repeated intrathecal treatments with LPA1 antagonist, AM966, which did not show acute action. All these results suggest that IPS model is an experimental animal model, which mimics the pathophysiology and pharmacotherapy in fibromyalgia in clinic, and LPA1 signaling plays crucial roles in the IPS-induced fibromyalgia-like abnormal pain.

## Highlights

• Intermittent psychological stress-induced fibromyalgia-like mouse model.
• Female-predominant gender difference after the gonadectomy.
• Pharmacotherapeutical characteristics similar to clinical evidence for fibromyalgia.
• Complete loss of abnormal pain in lysophosphatidic acid receptor 1 knockout mice.
• Complete blockade of established pain by repeated treatments of LPA antagonist.

## Introduction

Since animal models of neuropathic pain have been developed (Bennett and Xie, 1988, Ossipov and Porreca, 2013), much effort has been devoted to clarifying the mechanisms towards discovery of drug to treat neuropathic pain, by using physiological, anatomical (Basbaum et al., 2009, Devor, 2013) and molecular biological analyses (Ueda, 2006, Ueda, 2008, Costigan et al., 2009, Kuner, 2010, Hill, 2013). On the other hand, little is known of details of central pain, which is closely related to the emotional disturbance, since it is poorly characterized in terms of causes, primary and secondary loci. Fibromyalgia, a representative central and generalized pain is known to comprise an approximately 2% population ratio in developed countries (Russell et al., 1992, Clauw, 2014). Fibromyalgia patients are less responsive to classic and commonly used analgesic regimens, such as nonsteroidal anti-inflammatory drugs (NSAIDs), opioids and surgery. Although there is an increasing body of evidence supporting a role for peripheral small fiber neuropathy as well as central mechanisms as a causative factor in fibromyalgia (Levine and Saperstein, 2015, Clauw, 2015, Doppler et al., 2015, Ramirez et al., 2015), limited etiological information of fibromyalgia delays the reasonable diagnosis and therapy. Therefore, basic studies using animal models, which mimic pathophysiological symptoms and pharmacotherapeutical sensitivities in fibromyalgia patients, would be expected for advancing diagnosis or therapy.

The pioneering works by Levine’s group has demonstrated that vagotomized animals (Khasar et al., 1998b) show widespread pain (Khasar et al., 1998a, Chen et al., 2008, Furuta et al., 2009) and its gender difference (Levine et al., 2006), as seen in FM patients (Clauw, 2014). The pharmacotherapeutical study revealed that vagotomy-induced hyperalgesia is sensitive to antidepressants, gabapentinoids, and morphine (Furuta et al., 2009). The acid saline-induced muscle (ASM) pain model (Sluka et al., 2001), which shows chronic and widespread pain, has been frequently discussed as a fibromyalgia-like chronic muscle pain. The pharmacotherapeutical studies revealed that model animals are responsive to morphine (Sluka et al., 2002) as well as antidepressants and gabapentinoids (Yokoyama et al., 2007, Kim et al., 2009, DeSantana et al., 2013). Based on pharmacotherapeutical findings, reserpine-induced biogenic amine depletion model has been also proposed as a fibromyalgia-like pain model (Nagakura et al., 2009). Animals repeatedly treated with reserpine are responsive to several antidepressants and pregabalin (Hoppa et al., 2012), but it remains to be determined whether or not this model shows gender difference. On the other hand, we have established intermittent cold stress (ICS) model, which shares many clinical features, such as chronic, widespread, female-dominant pain (Nishiyori and Ueda, 2008). Moreover, consistent with clinical observations (Clauw, 2014), ICS-exposed mice are resistant to systemically and centrally administrated morphine, concomitantly with a lack of activation of descending serotonergic pain inhibitory pathway (Nishiyori et al., 2010). Indeed, the intrathecal injections of antidepressant agents, which mainly inhibit re-uptake of monoamines and cure ICS-induced thermal and mechanical hyperalgesia (Nishiyori et al., 2011). These findings are consistent with previous evidence that serotonin (5-HT), noradrenaline (NA), and their metabolites are less abundant in spinal CSF of FM patients (Russell et al., 1992).

From the point of view how brain mechanisms are involved in fibromyalgia or fibromyalgia-like pain model, we developed a novel intermittent psychological stress (IPS)-induced fibromyalgia-like pain model in mice on the analogy of ICS model, since ICS model may include the influence of altered peripheral or autonomic nervous system. In the present study, we revealed that the IPS-induced pain model mimics the ICS-induced pain model in terms of pathophysiology and pharmacotherapy, but differs from neuropathic pain (NP) model in many aspects of pathophysiology and pharmacotherapy. The present study, however, also revealed that the involvement of lysophosphatidic acid (LPA) receptor 1 system is common in various fibromyalgia-like generalized chronic pain and NP models.

## Material and methods

### Animals

Male and female C57BL/6J mice (TEXAM Corporation, Nagasaki, Japan) and homozygous mutant mice for the LPA1 receptor gene (LPA1^−/−^), weighing 20–25 g were used. They were kept in a room maintained at 21 ± 2 °C and 55 ± 5% relative humidity with a 12 h light/dark cycle, and had free access to a standard laboratory diet and tap water. All procedures used in this work were approved by the Nagasaki University Animal Care Committee, and complied with the fundamental guidelines for the proper conduct of animal experiments and related activities in academic research institutions under the jurisdiction of the Ministry of Education, Culture, Sports, Science and Technology, Japan.

### Drug treatments

Drugs used were administered through intraplantar (i.pl., 20 μl), subcutaneous (s.c., 100 μl/10 g), intraperitoneal (i.p., 100 μl/10 g), intracerebroventricular (i.c.v., 5 μl) or intrathecal (i.t., 5 μl) routes. The i.t. injection was given into the space between spinal L5 and L6 segments, according to the method described by Hylden and Wilcox (1980). Diclofenac purchased from Wako (Osaka, Japan) was dissolved in physiological saline for i.p. injection. Morphine hydrochloride (Takeda Chemical Industries, Osaka, Japan) was dissolved in the physiological saline for s.c. injection or in the aCSF (125 mM NaCl, 3.8 mM KCl, 1.2 mM KH_2_PO_4_, 26 mM NaHCO_3_, 10 mM glucose, pH 7.4) for i.c.v. injection. Pregabalin. mirtazapine and duloxetine hydrochloride were kindly provided by Meiji Seika Pharma Co., Ltd. (Kanagawa, Japan). Pregabalin was dissolved in physiological saline for i.p. injection or in aCSF for i.c.v. or i.t. injection. Mirtazapine was dissolved with 0.5% carboxymethylcellulose in physiological saline solution for i.p. injection or further diluted with aCSF for i.c.v. or i.t. injection. Duloxetine hydrochloride was dissolved in 0.5% dimethylsulfoxide (DMSO) for i.p. injection, while for the central injections, it was first dissolved in 300 mg/ml and then diluted by aCSF. LPA1 receptor antagonist, AM966 (2-[4-[4-[4-[[(1R)-1-(2-chlorophenyl)ethoxy]carbonylamino]-3-methyl-1,2-oxazol-5-yl]phenyl]phenyl]acetic acid) was first dissolved in 100 mM DMSO and diluted with aCSF for i.c.v. or i.t. injection.

### Gonadectomy

Six-week-old male and female mice were gonadectomized by removing ovaries or testes, respectively under pentobarbital (50 mg/kg i.p.) anesthesia. After the surgery, mice were kept in a soft bed cage with some food inside so that the animals could feed themselves without difficulty in standing. Gonadectomized mice were used for further stress treatments and nociception tests 3 weeks later.

### Intermittent psychological stress-induced pain model

**Fig. 1:**
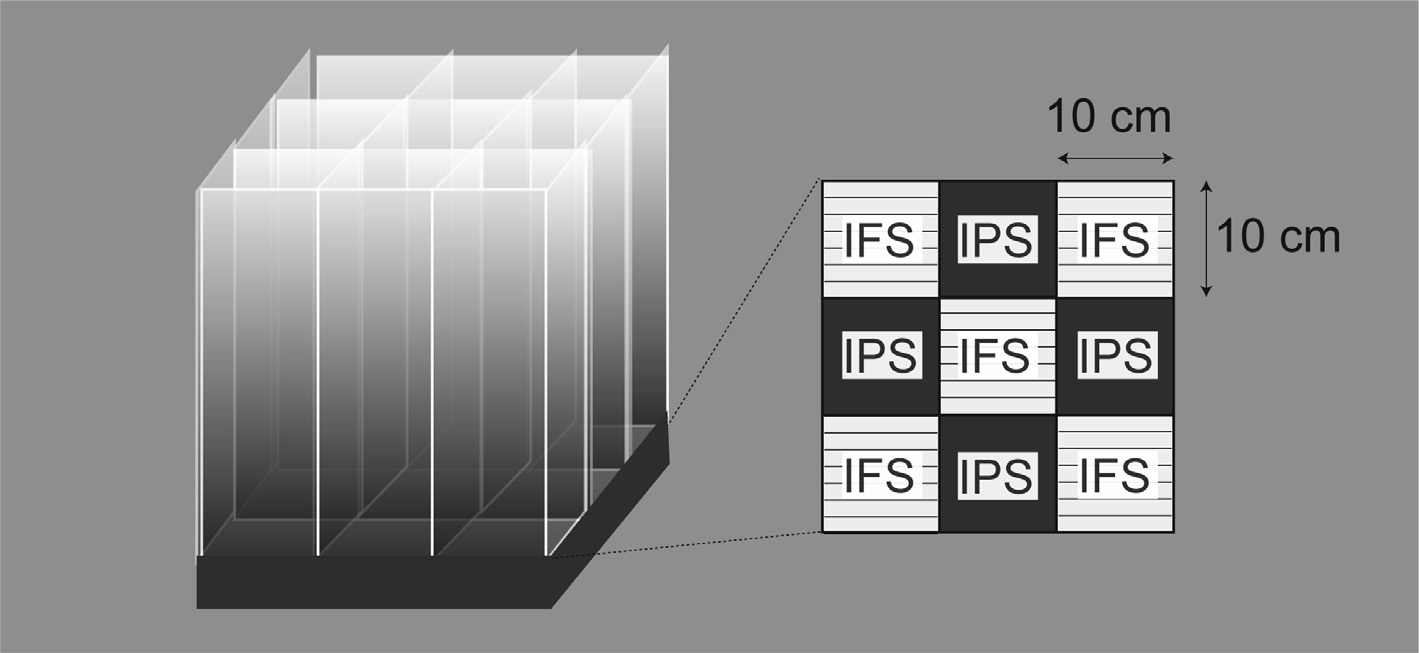
Schematic illustration of the protocol of intermittent psychological stress exposure. Communication box has nine compartments (10 cm × 10 cm) divided transparent Plexiglas walls. For the repeated foot stress exposure, 120-times of electrical shock (0.6 mA, 1 s duration) was delivered with every 47 s halt through the grid floor by a shock generator, while for the intermittent stress exposure, 120-times of electrical shock (0.6 mA, 1 s) was randomly delivered by the program of cycler timer, CBX-CT. Details are described under the Material and methods. For the psychological stress, mice were put on the floors covered with plastic plates.

Mice were exposed to intermittent psychological stress, by using the communication box (CBX-9M, Muromachi-Kikai, Tokyo, Japan) that has nine compartments (10 cm × 10 cm) divided transparent plastic walls. Physical stress was given mice through the grid floor by a shock generator (CSG-001, Muromachi-Kikai, Tokyo, Japan) and cycler timer (CBX-CT, Muromachi-Kikai, Tokyo, Japan), as elsewhere reported (Shimoda et al., 2010, Murata et al., 2017). For the repeated physical stress, short duration of electrical foot-shock (0.6 mA, 1 s) was delivered with every 47 s halt (120 times), while for the intermittent physical stress, the same number of shock was delivered randomly, taking 24–96 min. Both intermittent and repeated foot-shock stress, mice were individually put in the compartment located at the center and 4 corners without cover (Fig. 1). For the intermittent and repeated psychological stress, on the other hand, mice were put in remaining 4 compartments with insulating plastic cover. Mice that were exposed to psychological stress but not physical stress could see, hear, and smell the foot-shocked mice, despite the communication box is covered by a lid. In all experimental paradigms, the stress was given once per day for 5 days and we call the post-stress day 1 as P1.

### Intermittent cold stress-induced pain model

Mice were exposed to ICS, as described reported (Nishiyori and Ueda, 2008). Briefly, for the ICS model, mice were placed in a cold room at 4 °C overnight (from 4:30 pm to 10:00 am), followed by ICS with alternating environmental temperatures between 24 and 4 °C every 30 min from 10:00 am to 4:30 pm. These procedures were repeated twice. On day 3, the mice were returned to and adapted to the room at 24 °C for 1 h before the behavior tests. We designated day 3 following the onset of stress exposure as day 1 post-stress exposure (P1). Mice in the control group were kept at 24 °C for all 3 days (from 4:30 pm on day 1 to 10:00 am on day 3). During the stress period, two mice were kept in each cage (12 × 15 × 10.5 cm), with free access to food and agar in place of fluid.

### Repeated acid saline-induced muscle pain model

Mice were injected 20 μl of pH 4.0 sterile saline into the right gastrocnemius muscle 5 days apart to induce generalized chronic muscle pain, as reported by Sluka et al. (2003).

### Inflammatory pain model

The pain model was produced by an i.pl. injection of 20 μl aliquot of 1 mg/ml of complete Freund’s adjuvant (CFA, Wako, Osaka, Japan).

### Mechanical paw pressure test

Mice were placed in a plexiglass chamber on a 6 × 6 mm wire mesh grid floor and allowed to acclimatize for a period of 1 h, as reported (Uchida et al., 2010). A mechanical pain stimulus was then delivered to the middle of the plantar surface of the right hind paw using Electronic digital von Frey Anesthesiometer and Rigid Tip (Model 2390, 90 gram probe; IITC Inc., Woodland Hills, CA, USA). The pressure needed to induce a flexor response was defined as the pain threshold. A cut-off pressure of 20 g was set to avoid tissue damage.

### Thermal paw withdrawal test

The thermal pain threshold was evaluated by the latency of paw withdrawal upon a thermal stimulus (Hargreaves et al., 1988, Uchida et al., 2010). Unanesthetized animals were placed in plexiglass cages on top of a glass sheet, and an adaptation period of 1 h was allowed. The thermal stimulator (IITC Inc., Woodland Hills, CA, USA) was positioned under the glass sheet and the focus of the projection bulb was aimed exactly at the middle of the plantar surface of the animal. A mirror attached to the stimulator permitted visualization of the plantar surface. A cut-off time of 20 s was set to prevent tissue damage.

### Mechanical muscle pain test using pressure analgesia meter

Mechanical muscle pain test was performed by use of Randall-Selitto-type pressure analgesia meter MK-201D (Muromachi Kikai, Tokyo, Japan), which displays the pressure digitally. For the evaluation of muscle pain threshold, the pressure was given to the right femur and the threshold to cause the struggling behavior was measured.

### Acetic acid-induced writhing test

The number of typical writhing behaviors after intraperitoneal injection of 0.9% acetic acid solution (0.1 ml per 10 g body weight) was counted for 20 min post-injection.

### Intra-colon capsaicin test

A visceral pain model was carried out by a slightly modified method of Laird et al., 2001, Laird et al., 2002. Capsaicin was dissolved in 10% ethanol and 10% Tween-80 in physiological saline. A 20 μl aliquot of 0.3% capsaicin was injected into the colon, and the licking of the abdomen were measured for 20 min.

### Tail suspension test

Experiments were carried out, as reported (Sasaki et al., 2015, Ueda, 2017). Mice were suspended 30 cm above the floor using adhesive tape for a 6-min session. The total duration of immobility in the last 4-min period was evaluated.

### Forced swimming test

Experiments were carried out, as reported (Sasaki et al., 2015, Ueda, 2017). Mice were gently released into a transparent Plexiglas cylinder (20 cm in height, 10 cm in diameter) filled with water (23 °C) up to a height of 7.5 cm for a 6-min session. The total duration of immobility in the last 4-min period was evaluated.

### Plasma corticosterone measurement

Mouse plasma samples (20 μl) were diluted with 180 μl enzyme immunoassay (EIA) buffer to prepare 1/10 plasma sample. Following procedures for the measurement of plasma corticosterone levels were performed by the manufacture’s protocol of Corticosterone EIA Kit (Cayman, ITEM No. 500655, Lot. 0457099, Ann Arbor, MI).

### Monoamine turnover rate measurement

The samples of spinal dorsal horn (dorsal half) at the lumber 4–6 levels of spinal cord were homogenized in 0.2 m perchloric acid containing 0.1 mm EDTA and centrifuged for 15 min at 20,000*g* at 0 °C. The supernatant was then filtered through 0.22 μm polyvinylidene fluoride (PVDF) micropore filters (Millipore), and the filtrate was analyzed by high-pressure liquid chromatography (HPLC) coupled to an electrochemical detection system (graphite electrode *vs.* Ag/AgCL reference, Eicom). Briefly, a Prepak AC-ODS 4.0 × 5.0 mm precolumn and an Eicompak SC-5ODS 3.0 × 150 mm column were used for separations; the perfusate was a mobile phase consisting of 100 mm citrate, 100 mm sodium acetate, 15% methanol, 190 mg/L sodium 1-octanesulfonate, and 5 mg/L EDTA, adjusted to pH 3.5 using glacial acetic acid and pumped at a rate of 0.5 ml/min. The working electrode (WE-3G) potential was set at +0.75 V. The column temperature was maintained at 25 °C. All analyte information, including the retention times, peak heights, concentrations, and recovery rate of the internal standards, were calculated in relation to standard curves generated for known concentrations of external standards run daily. In the present study, monoamines and their metabolites measured were dopamine (DA), NA, 5-HT, 3,4-dihydroxyphenylacetic acid (DOPAC), homovanillic acid (HVA), 3-methoxy-4-hydroxyphenylglycol (MHPG), 5-hydroxyindoleacetic acid (5-HIAA).

### Statistical analysis

Data were analyzed using the Student’s *t*-test, one-way analysis of variance with Tukey’s multiple comparisons test or two-way ANOVA followed by Tukey’s multiple comparisons test. The criterion of significance was set at p < 0.05. All results are expressed as means ± standard error of the mean (SEM).

## Results

### Chronic and bilateral abnormal pain by intermittent psychological stress

To produce foot-shock and psychological stress, we used the communication box that has nine compartments divided transparent plastic walls (Fig. 1), and details were described under Material and methods. Five mice were subjected to electrical foot shocks, while four mice were exposed to psychological stress in the compartments, in which the grid floors were covered with insulating plastic plates. Mice given with randomly programmed electrical shock (intermittent foot-shock stress/IFS) showed nociceptive behaviors, such as rearing, jumping and vocalization upon each electrical footshock. Other four mice, on the other hand, are supposed to keep watching and smelling the foot-shocked mice, and listening to their vocalization over the plexiglass. The mice without foot shock (intermittent psychological stress/IPS) looked restless without significant behavioral abnormalities throughout the foot-shock or psychological stress paradigm. Similar nociceptive responses and less significant behavioral changes were also observed when repeated foot-shock stress/RFS or repeated psychological stress/RPS, respectively.

**Fig. 2:**
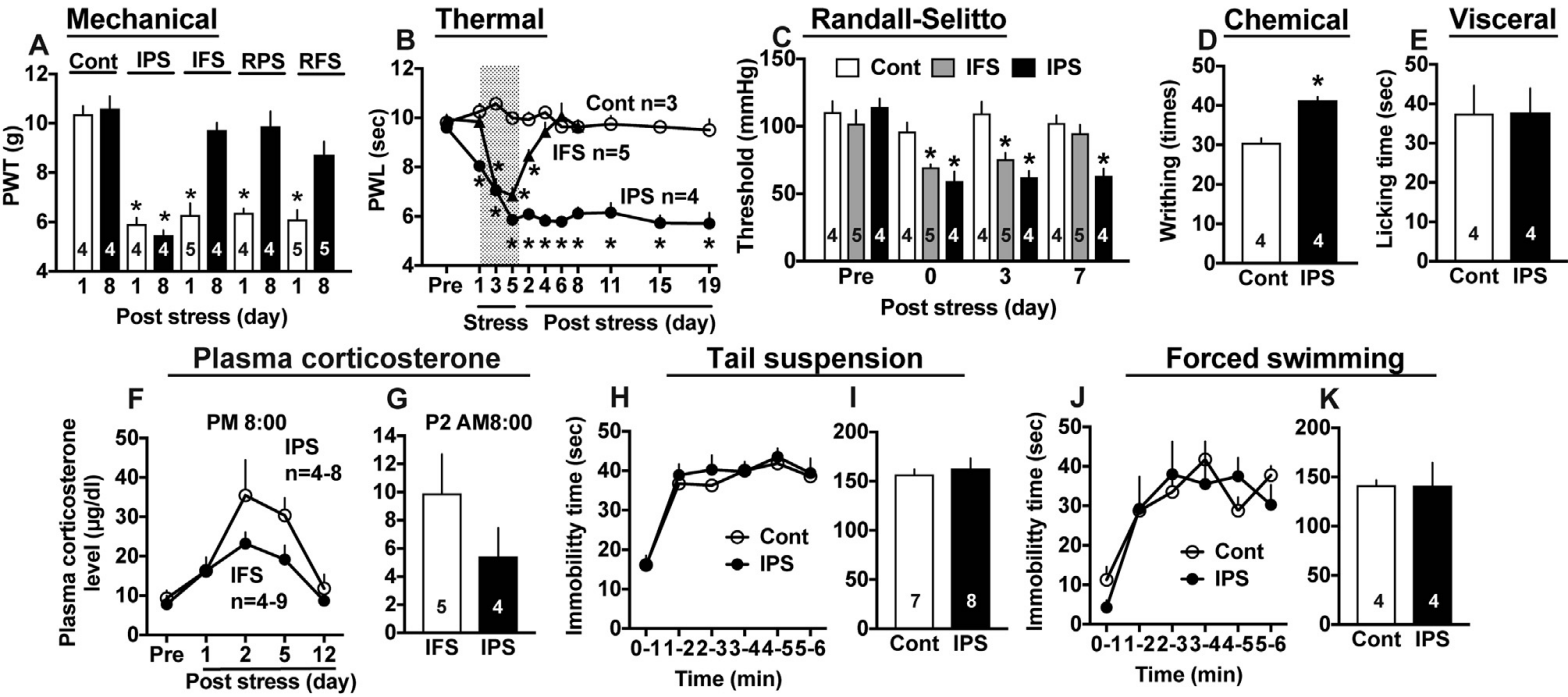
Intermittent psychological stress causes chronic and bilateral hyperalgesia. (A) The development of chronic pain in the paw pressure (mechanical nociception) test after intermittent psychological stress (IPS), but not after intermittent foot shock (IFS), repeated psychological stress (RPS) or repeated foot shock stress (RFS). (B) The development of long-lasting thermal hyperalgesia after IPS, but not after IFS. (C) Mechanical muscle pain in Randall-Selitto test. (D, E) Chemical (somatic) hyperalgesia in the acetic acid-induced writhing test (D), but no change in the visceral pain induced by intra-colon capsaicin injection at P5 after IFS treatment (E). (F, G) Daily variation of plasma corticosterone levels at PM8:00 (F) and the plasma levels at AM8:00 of P2 (G) after IFS or IPS. (H-K) No evidence for depression-like behaviors by IPS in the tail suspension (H,I) and forced swimming (J,K) tests. Results represent the time course (H,J) and total time (I,K) of immobility time. The number of animals used is indicated in each column (A, C, D, E, G, I, K). (A) ^*^p < 0.05, *vs.* Cont, one-way ANOVA followed by Tukey’s multiple comparisons test, (B, C, F, H, J) ^*^p < 0.05, *vs.* Cont, two-way ANOVA followed by Tukey’s multiple comparisons test, (D, E, G, I, K) ^*^p < 0.05, Cont *vs.* IPS, Student’s *t*-test, (F) IFS *vs.* IPS, Student’s *t-*test.

When the mechanical nociception test is performed, the basal threshold of untreated control mice was 10.37 ± 0.33 g (n = 4), and 10.6 ± 0.5 g (n = 4) at day 1 and day 8. Both thresholds were markedly reduced to 5.92 ± 0.25 g (n = 4) and 5.47 ± 0.2 g (n = 4) , respectively by IPS, as shown in Fig.2A. Mechanical hyperalgesia at day 8 was not observed when the stress was replaced by IFS, RPS or RFS, which just caused transient mechanical hyperalgesia at P1. The IPS-induced thermal hyperalgesia was observed at as early as 24 h after the initial psychological stress (day 1), and peak hyperalgesia was observed at day 5 and later even at post stress day 19 (P19), as shown in Fig.2B. On the other hand, IFS also caused significant hyperalgesia at day 3, 5 and P2, but it is getting recovered to the normal level at P6. In the mechanical muscle pain test, the basal threshold was 110.5 ± 0.8 mmHg (n = 4). As shown in Fig.2C, the pain threshold of IFS- and IPS-treated mice was significantly decreased at P0 and P3. At P7, the hyperalgesia was still observed in IPS-mice, while no significant change was observed in IFS-mice. There was also observed a somatic hyperalgesia in the acetic acid (i.p.)-induced writhing test (Fig.2D), while no significant visceral pain was observed in the capsaicin (i.colon)-induced pain behavior (Fig.2E).

For the purpose to examine the relationship of endocrine system to the differential persistency in abnormal pain between IFS and IPS, we attempted to measure the plasma levels of corticosterone. As the corticosterone levels in mice with IFS were higher than those with IPS at PM 8:00 and AM 8:00, however, there was observed no evidence that the elevated stress-induced endocrine system causes long-lasting abnormal pain (Fig.2F, G).

Regarding the depression-related behaviors, IPS-treated mice showed no increase in the immobility time in the tail-suspension test throughout 6 h (Fig.2H, I), but not in the forced swimming test (Fig.2J, K).

### Gender difference

**Fig. 3:**
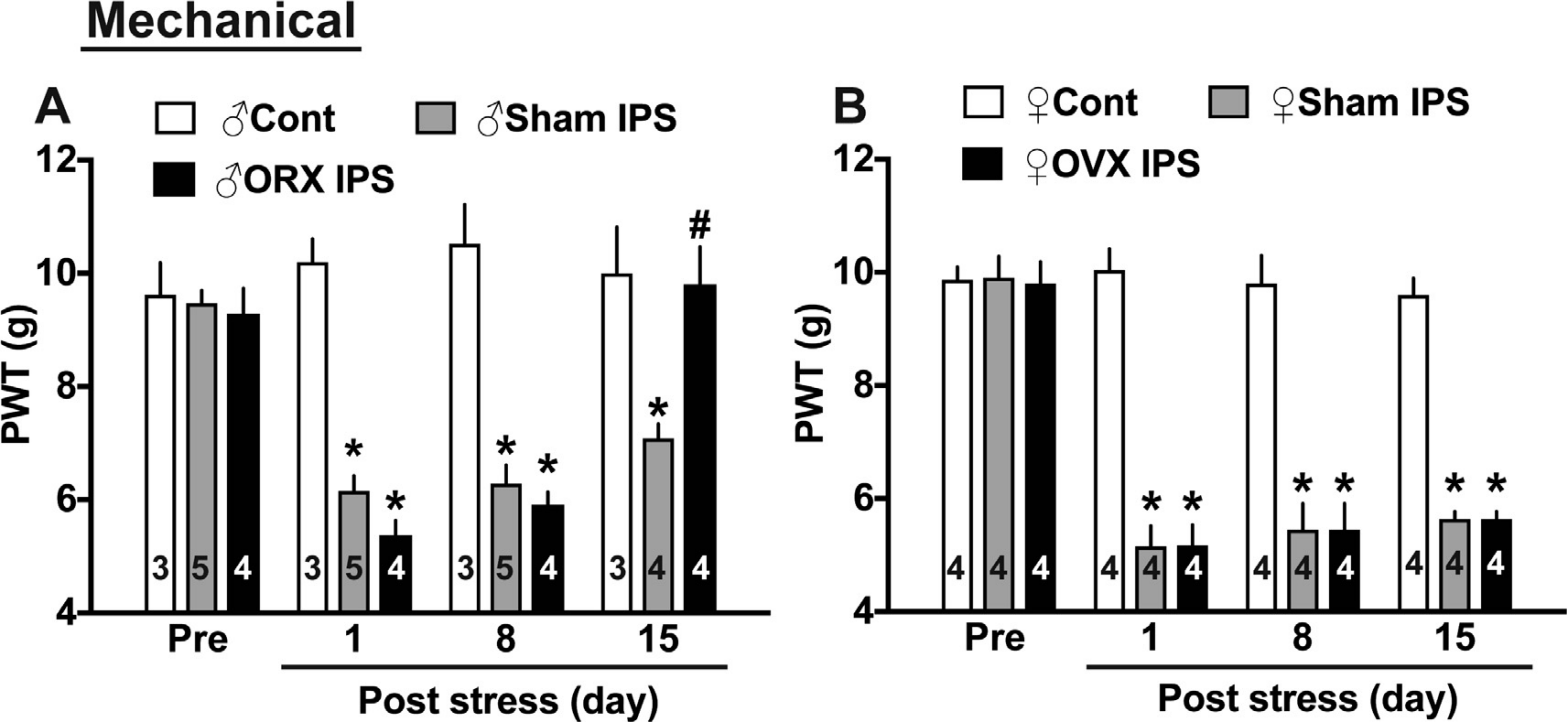
Gender difference in the effects of gonadectomy on IPS-induced mechanical hyperalgesia. (A) Gradual recovery of hyperalgesia in IPS-treated male mice with and without gonadectomy (removal of testes). (B) Stable long-lasting hyperalgesia in IPS-treated female mice with and without gonadectomy (removal of ovaries). ^*^p < 0.05, *vs.* control mice, two-way ANOVA followed by Tukey’s multiple comparisons test, ^#^p < 0.05, ♂Sham IPS day15 v*s.* ♂ORX IPS day15, two-way ANOVA followed by Tukey’s multiple comparisons test.

Six-week-old female and male mice were gonadectomized by removing ovaries or testes, respectively. There was no significant gender difference in the basal nociceptive threshold (indicated as ‘Pre’ in the figure) and no difference in the nociceptive threshold between sham operation or gonadectomy in both genders 3 weeks later (Fig.3A,B). Following the IPS, significant mechanical hyperalgesia was observed in both genders. The levels in male mice were decreased from 9.47 ± 0.23 g to approximately 6 g at P1, 7 g at P15, while those in female mice to approximately 5 g at P1-15 from 9.91 ± 0.37 g, respectively. These results suggest that female mice show a little more stable and intense abnormal pain than male mice. Female predominant gender difference became evident at P15 after the gonadectomy. Abnormal pain at P15 after IPS completely disappeared in orchidectomized male mice, but the same level of abnormal pain was still observed in ovariectomized female mice. As reported previously (Nishiyori and Ueda, 2008), gonadectomy had no significant influence on the gross behavioral activities.

### Turnover rate of spinal monoaminergic systems

**Fig. 4:**
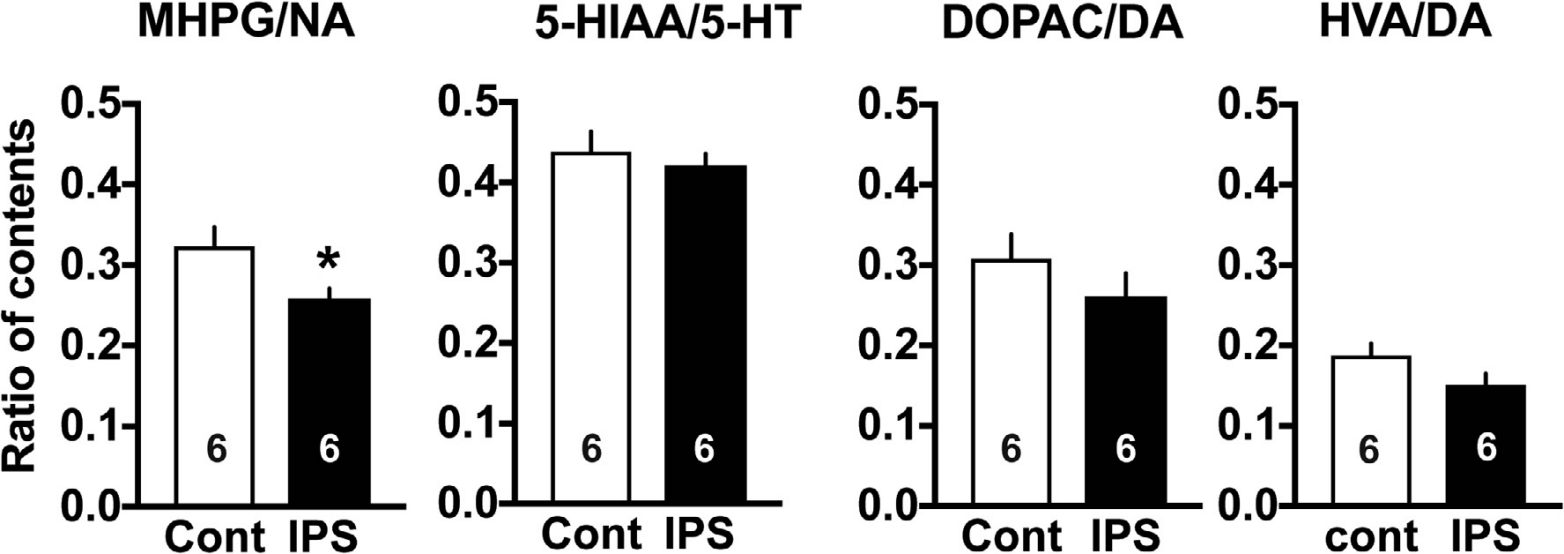
Turnover rates of spinal cord monoaminergic systems. (A) The turnover rate of noradrenaline (NA) represents the ratio of the contents of MHPG, a major metabolite to NA contents in the dorsal horn of spinal cord. (B, C) The turnover rate of dopamine (DA) represents the ratio of the contents of DOPAC (B) or HVA (C), major metabolites to DA contents. (D) The turnover rate of serotonin (5-HT). 5-HIAA is a major metabolite. ^*^p < 0.05, *vs.* control mice, Student’s *t*-test.

To examine the medulla-spinal descending or spinal monoaminergic activities, the ratio of metabolite(s) to neurotransmitter contents in the spinal dorsal horn was measured by use of high performance liquid chromatography with electrochemical detection. As shown in Fig. 4, only noradrenergic turnover rate was significantly decreased, though a weak, but not significant decrease was also detected in dopaminergic turnover rate.

### Lack of actions of diclofenac and morphine

**Fig. 5:**
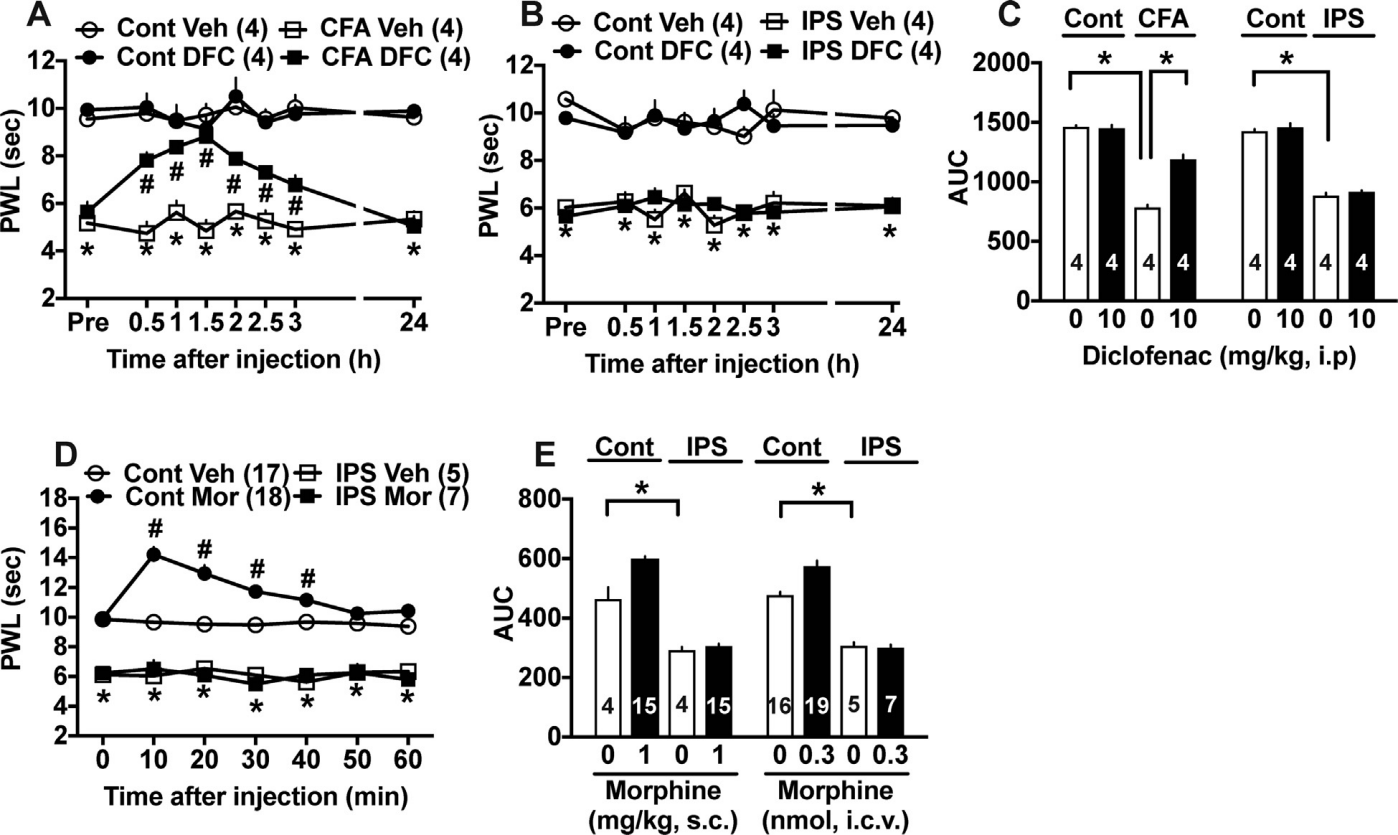
Lack of beneficial actions of diclofenac and morphine against thermal hyperalgesia. (A) Time course of anti-thermal hyperalgesia by diclofenac (10 mg/kg, i.p.) in the complete Freund’s Adjuvant (CFA)-induced inflammatory pain model. (B) Lack of beneficial effects of diclofenac in the IPS-model. (C) Quantitative comparison of diclofenac effects in both CFA- or IPS-models. Results represent the AUC for 3 h. (D) Lack of morphine analgesia in the IPS-model. Morphine (0.3 nmol, i.c.v.) showed potent analgesic effects in control mice, but not in IPS-treated mice. (E) Quantitative comparison of anti-nociceptive activities by morphine (s.c. and i.c.v.) in both control and IPS-model mice. Results represent the AUC for 1 h. (A, B, D) Two-way ANOVA followed by multiple comparisons test. (A) ^*^p < 0.05, *vs.* Cont Veh, ^#^p < 0.05, *vs.* CFA Veh, (B) ^*^p < 0.05, *vs.* Cont Veh, (D) ^*^p < 0.05, ^#^p < 0.05, *vs.* Cont Veh, (C, E) ^*^p < 0.05, one-way ANOVA followed by multiple comparisons test.

In the thermal nociception test, 10 mg/kg (i.p.) of diclofenac, an NSAIDs was given at day 3 after the treatment with complete Freund’s adjuvant (CFA), as shown in Fig.5A. Diclofenac showed potent anti-hyperalgesic actions with peak effects at 1.5 h and lasted for 3 h, but not at 24 h. However, the same dose of diclofenac did not show any beneficial effects at P5 in the IPS model (Fig.5B). Quantitative comparison by use of area under the curve (AUC) of anti-hyperalgesic effects supported the differential potencies of diclofenac between inflammatory and IPS models (Fig.5C).

When 0.3 nmol (i.c.v.) of morphine was given, significant analgesia was observed for 40 min in control mice, while no analgesia was observed throughout 60 min in IPS-treated mice (Fig.5D). The lack of morphine analgesia was also observed when it was given subcutaneously (Fig.5E).

### Brain-specific beneficial actions of pregabalin

**Fig. 6:**
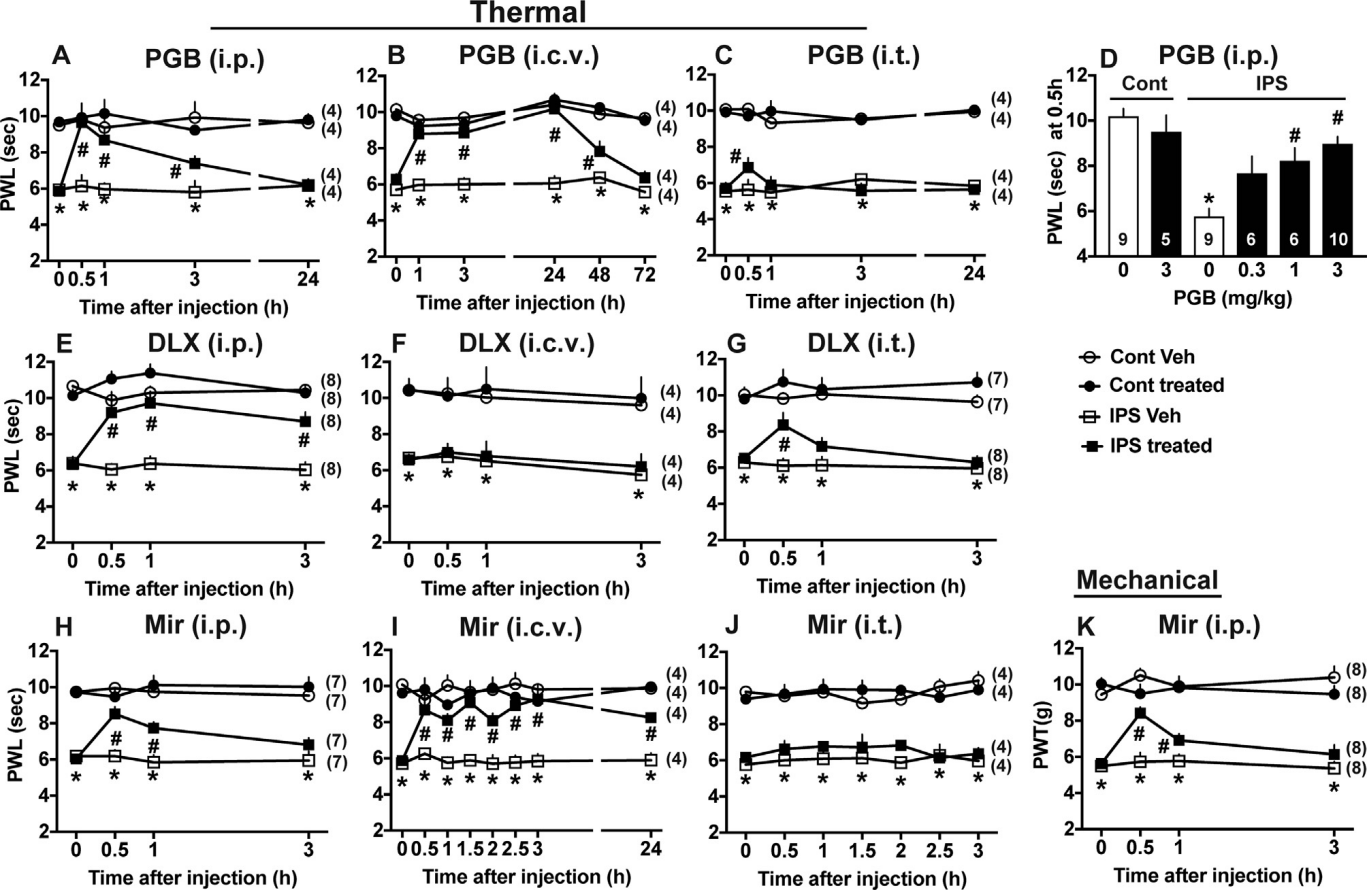
Beneficial actions of pregabalin, duloxetine and mirtazapine in IPS-treated mice. (A-C) Time course of PGB-induced anti-hyperalgesia at 3 mg/kg i.p. (A), 0.3 μg i.c.v. (B) or 3 μg i.t. (C). (D) Dose-dependent reversal of thermal hyperalgesia by PGB (i.p.). (E-G) Time course of duloxetine (DLX) effects at 30 mg/kg i.p. (E), 3 μg i.c.v. (F) or 1 μg i.t. (G). (H-J) Time course of mirtazapine (Mir) effects at 1 mg/kg i.p. (H), 1 μg i.c.v. (I) or 1 μg i.t. (J). (A-J) Results represent the threshold in the thermal nociception test. (K) Time course of mirtazapine (1 mg/kg, i.p.) in the paw pressure test. (A-C, E-K) ^*^p < 0.05, *vs.* IPS, ^#^p < 0.05, test drug (PGB, DLX or Mir) *vs.* vehicle (Veh), two-way ANOVA followed by Tukey’s multiple comparisons test, (D) ^*^p < 0.05, *vs.* Cont (0), ^#^p < 0.05, *vs.* IPS (0), one-way ANOVA followed by multiple comparisons test.

As shown in Fig.6A, the single intraperitoneal (i.p.) injection of pregabalin (PGB) at 3 mg/kg showed potent anti-hyperalgesic effects with peak effects at 0.5 h. Significant beneficial actions last for 3 h, but no significant actions were detected at 24 h. The i.c.v. injection of PGB at as low as 0.3 μg showed very long-lasting anti-hyperalgesic effects for 48 h were observed (Fig.6B), while very weak and transient beneficial effects were detected by i.t. injection of 3 μg PGB (Fig.6C). From the dose-related effects using 0.3, 1 and 3 mg/kg (i.p.), the ED50 at 0.5 h was calculated as approximately 1 mg/kg (Fig.6D).

### Differential sites of beneficial actions of duloxetine and mirtazapine

The systemic injection of duloxetine (DLX), a serotonin-noradrenalin re-uptake inhibitor-type antidepressant (Iyengar et al., 2004) at 30 mg/kg (i.p.) showed potent anti-hyperalgesic effects with peak effects at 1 h (Fig.6E). Although no significant beneficial effects were observed by 3 μg i.c.v. of DLX (Fig.6F), but significant anti-hyperalgesic effects were observed by 1 μg i.t. of DLX (Fig.6G). In contrast, mirtazapine (Mir), a novel type of antidepressant (Millan et al., 2000, Nutt, 1997) at 1 mg/kg i.p. showed significant anti-hyperalgesic effects at 0.5 and 1 h, but not 3 h in the thermal nociception test (Fig.6H). When 1 μg Mir was given through an i.c.v. route, very potent and long-lasting anti-hyperalgesic effects for more than 24 h were observed (Fig.6I), while no significant beneficial effects were observed with 1 μg (i.t.) of Mir (Fig.6J). Significant beneficial effects with Mir (1 mg/kg, i.p.) were also observed in the paw pressure test (Fig.6K).

### Involvement of LPA1 receptor signal in various fibromyalgia-like pain models

**Fig. 7:**
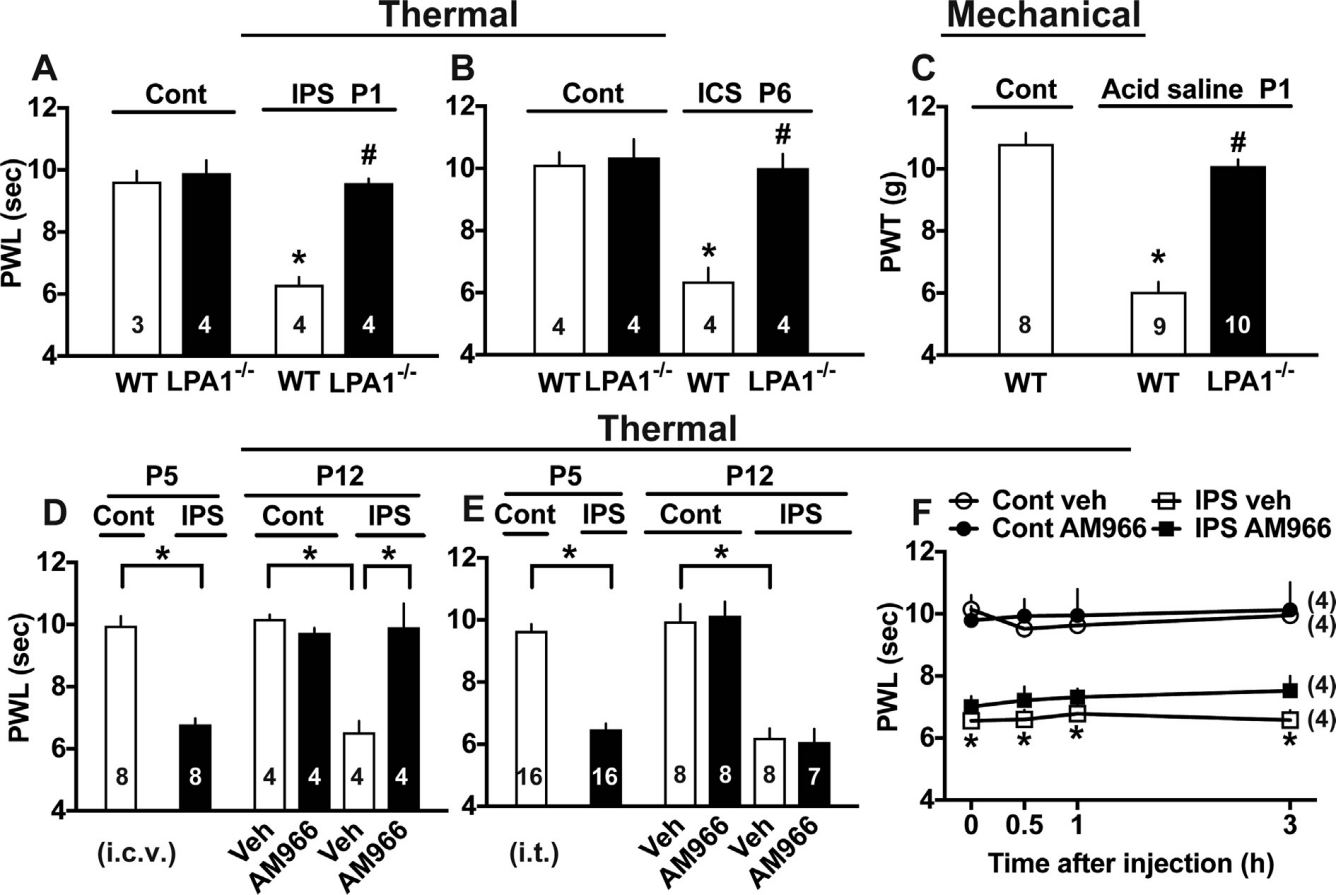
Involvements of LPA1 signaling in various fibromyalgia-like pain models. (A-C) Lack of abnormal pain in LPA1^−/−^ mice in IPS (A), intermittent cold stress/ICS (B) or acid saline-induced chronic pain (C) model. Results represent the nociceptive threshold in the thermal nociception test (A, B) and in the paw pressure test (C). (D, E) Therapeutical effects of daily treatments of vehicle (Veh) or LPA1 antagonist AM966 (1 μg i.c.v. or 3 μg i.t.) from P5 to P11 in IPS-treated mice. Results at P5 represent the basal nociceptive threshold before the AM966 treatment in the thermal nociception test. Results at P12 represent the threshold at 24 h after the last injection at P11. (F) No significant effects by Veh or AM966 (1 μg i.c.v.) in control and IPS-treated mice at P5. (A-C) ^*^p < 0.05, *vs.* untreated control WT mice. ^#^p < 0.05, WT *vs.* LPA^−/−^ mice in each model, one-way ANOVA followed by Tukey’s multiple comparisons test. (D, E) ^*^p < 0.05, one-way ANOVA followed by multiple comparisons test. (F) ^*^p < 0.05 Cont veh *vs.* IPS AM966, two-way ANOVA followed by multiple comparisons test.

As shown in Fig.7A, the IPS-induced thermal hyperalgesia at P1 was completely abolished in mice deficient of LPA1 receptor gene. Similar complete blockade of intermittent cold stress (ICS)-induced thermal hyperalgesia at P6 (Fig.7B) or acid saline-induced mechanical hyperalgesia at P1 (Fig.7C) was observed in mice deficient of LPA1 receptor gene. When 1 nmol of AM966, a specific LPA1 receptor antagonist (Swaney et al., 2010) was given i.c.v. daily from P5 to P11, the thermal hyperalgesia was completely reversed at P12 (Fig.7D), while no significant change was observed when 3 nmol i.t. of AM966 was given (Fig.7E). However, 1 nmol of AM966 (i.c.v.) had no acute effects throughout 3 h in control mice or IPS-treated mice at P5 (Fig.7F).

## Discussion

The first issue in the present study is the development of intermittent psychological stress (IPS)-induced generalized pain model in mice. The IPS-treated mice showed long-lasting abnormal pain behaviors in the mechanical, thermal, muscle and somatic chemical nociception tests, though they did not show any hypersensitivity in the visceral pain behaviors induced by capsaicin (i.colon). Unique property is observed in the finding that the persistent pain lasting over 19 days was observed when IPS was given to mice, while transient pain lasting within 6 days was observed when intermittent foot shock (IFS), repeated psychological (RPS) and repeated foot shock stress (RFS) were given. These findings suggest that randomly programmed intermittent or unexpected stress causes more stable pain behaviors than simply repeated or physical stress, though underlying mechanisms remain elusive. It is interesting to assume that IPS-treated mice may experience empathy for the mice given painful physical stress, as previously discussed (Langford et al., 2006).

We have previously reported the development of another type of generalized pain model in mice, which is caused by intermittent cold stress or ICS (Nishiyori and Ueda, 2008). The nature of IPS-model is in principle very similar to the ICS-model in terms of pathophysiology and pharmacotherapeutic potentials (Nishiyori and Ueda, 2008, Nishiyori et al., 2010, Nishiyori et al., 2011). In the present study using IPS, we further demonstrated the hyperalgesia in somatic pain to chemical stimuli by intraperitoneal injection of acetic acid, and in mechanical muscle pain to the femur. In the pathophysiology, as well as the wide spread generalized pain, a unique gender difference was observed in the IPS-model. IPS-induced chronic pain remained in ovariectomized female mice at P15, while the pain threshold in orchidectomized male mice at P15 returned to the normal level. Similar female-specific gender difference after gonadectomy was also observed in the ICS model, though no or very little difference of hyperalgesia was observed in both models without gonadectomy (Nishiyori and Ueda, 2008). The gender difference of gradual recovery of pain threshold after the IPS in gonadectomized mice may be explained by the possibilities that female mice may possess a potency in nature to retain pain memory for long periods, or that pain-related roles of sex hormone generating organ may differ between genders. Recent clinical evidence describes that fibromyalgia is more common in premenopausal women with climacteric symptoms than in postmenopausal ones without them (Carranza-Lira and Hernandez, 2014), being not inconsistent to the present finding that the gonadectomy did not affect the female IPS-induced pain. It should be more interesting subject to elucidate what mechanisms are involved in the recovery of IPS-induced pain in male mice.

Mice with IPS-induced pain showed resistance to diclofenac, a representative NSAIDs, which effectively blocked the CFA-induced inflammatory pain. They also showed resistance to morphine given i.c.v. or s.c. These negative results are in a good accordance to the case with fibromyalgia patients in clinic. There are several reports about the possible mechanisms underlying the lack of morphine or opioid analgesia (Sorensen et al., 1995, Julien et al., 2005; Rao and Clauw, 2004). Recent study revealed that the pain intensity of fibromyalgia patients is negatively correlated to the decreased pain-evoked neural activation in the nucleus accumbens in fMRI analysis reflecting opioid action or to the opioid binding in PET analysis (Harris et al., 2007, Schrepf et al., 2016). As it is well known that stress-induced analgesia is mediated by endogenous opioids (Butler and Finn, 2009), it is plausible that intense and repeated stress may cause excess release of endogenous opioids, which in turn lead to an analgesic tolerance to endogenous opioids. The decreased opioid action and opioid binding supposed from fMRI and PET studies of fibromyalgia patients may be related to the cellular desensitization of opioid receptor signaling, down regulation and possible counterbalance through anti-opioid mechanisms in neural circuits (Russell et al., 1992, Baraniuk et al., 2004, Inoue et al., 2003, Ueda, 2004). Alternative mechanisms underlying the lack of morphine analgesia may be related to the findings that fibromyalgia patients have decreased levels of spinal cord monoamines (Russell et al., 1992), which play roles in the descending pain inhibitory actions by morphine (Fields and Basbaum, 1978). We have recently observed that repeated treatments with small doses of donepezil, a representative medicine for Alzheimer completely cured the ICS-induced thermal and mechanical hyperalgesia, while did not recover the morphine analgesia (Mukae et al., 2015). All these results suggest that mechanisms underlying chronic pain and lack of morphine analgesia in the intermittent stress-induced pain model should be separately discussed.

Regarding monoamine systems, serotonin norepinephrine reuptake inhibitors (SNRIs) are more recommended for the treatment of fibromyalgia than selective serotonin reuptake inhibitors (Clauw, 2014). The present study also showed DLX, a representative SNRI has significant anti-hyperalgesic actions when given through i.t. route, but not through i.c.v. route. The measurement of turnover rate also supported this view that NA turnover rate in the spinal dorsal horn is significantly decreased, but not 5-HT turnover rate. The lack of change in 5-HT turnover rate in the IPS model is consistent to the case with the ICS model, though the ICS model showed a significant reduction of morphine (i.c.v.)-induced elevation of 5-HT turnover rate. As both IPS- and ICS-models caused no significant change on the depression-like behaviors, the anti-hyperalgesic actions of DLX seems to be relatively independent of antidepression effects. It is interesting to note that Mir has potent and long-lasting therapeutic actions when given through i.c.v., but not through i.t. route. These findings suggest that the site of Mir action for suppressing IPS-induced abnormal pain exists in the brain, but not in the spinal cord. It is reported that Mir has antagonist activities against adrenergic α2, 5-HT2, 5-HT3 and histamine H1 receptors, but does not have NA/5-HT reuptake inhibitor activity (Millan et al., 2000, Nutt, 1997). Thus, it seems that DLX and Mir share the common pain inhibitory pathways, though detailed mechanisms remain elusive.

PGB was also effective in treating the IPS-induced abnormal pain. The site of action of PGB seems to be in the brain, but not in the spinal cord or dorsal root ganglion, since PGB (i.c.v.) showed significant anti-hyperalgesia for more than 48 h, being consistent to the case (96 h) with gabapentin actions in the ICS-model. As the PGB (i.p.)-induced anti-thermal hyperalgesic actions in the present IPS model and anti-mechanical allodynic actions in the ICS model (Nishiyori and Ueda, 2008, Mukae et al., 2016) completely disappear within 24 h, the initial intense action of PGB (i.c.v.) may be enough to suppress unidentified feed-forward mechanisms underlying long-lasting abnormal pain in these IPS and ICS models, though additional key mechanisms for pregabalin or gabapentin other than calcium channel α2δ subunit may be also involved (Micheva et al., 2003, Bauer et al., 2009).

We have reported that lysophosphatidic acid (LPA), a representative lipid mediator and LPA1 receptor signaling initiate the neuropathic pain following partial sciatic nerve ligation of mice (Inoue et al., 2004, Ueda, 2006). This study revealed that LPA1 signaling is involved in the neuropathic demyelination of dorsal roots and upregulation of calcium channel α2δ1 and ephrinB1 gene, both which are closely related to hyperalgesia, respectively (Ueda, 2008, Ueda et al., 2013, Santos-Nogueira et al., 2015, Ahn et al., 2009). Recent studies further revealed that the LPA production initiated by synergistic activation of spinal cord neurons by pain transmitters, such as substance P and NMDA receptor agonist, is further self-amplified through an activation of microglial LPA receptors (Ueda, 2017, Velasco et al., 2017). Most recently clinical evidence demonstrates that LPA levels in the human synovial fluid are high as the pain severity due to osteoarthritis (McDougall et al., 2016). Accordingly, we speculated that the possible production of brain LPA following intense or synergistic neuronal activation may be involved in the abnormal pain production in several experimental fibromyalgia-like models. This speculation was proved by two different experiments in the present study, as follows; the IPS-, ICS- or acid saline-induced abnormal pain was all abolished in mice deficient of LPA1 gene, and repeated i.t. treatments with LPA1 antagonist AM966 (Swaney et al., 2010) from P5 to P11 completely reversed the established abnormal pain threshold to the normal level at P12, though AM966 has no acute action at P5. The latter evidence suggests the blockade of LPA1 receptor signaling could be expected for both prophylactic and therapeutic use. Taken altogether, antagonists against LPA receptors and various inhibitors of LPA-producing enzymes and related targets, which have good pharmacokinetic and pharmacodynamics properties, would be useful for the fibromyalgia therapy.

In conclusion, the present study revealed that the IPS model has some similarities to clinical evidence of fibromyalgia in terms of generalized chronic pain, a kind of female predominant gender difference only observed after gonadectomy and pharmacotherapeutical similarities using representative medicines. Furthermore, we found that LPA1 receptor signaling plays crucial roles in pathophysiology and pharmacotherapy. We also understand that it remains to be determined whether rodent models share the common nature and causes with fibromyalgia in clinic, and whether the intermittent stress-induced model is related to emerging mechanisms of peripheral small fiber neuropathy. However, we believe that further detailed pharmacotherapeutical studies in comparison to the clinical evidence would provide the validity of such rodent intermittent stress-induced pain models in future, and subsequent beneficial information in terms of fibromyalgia treatments.

## Disclosure statement

The authors have no disclosures relevant to the article to report.
